# Few-Shot Learning for Cytological Classification of Primary Lung Cancer and Pulmonary Metastases During Endobronchial Ultrasound

**DOI:** 10.3390/diagnostics16172790

**Published:** 2026-08-30

**Authors:** Ching-Kai Lin, Di-Chun Wei, Hsin-Hung Chou, I-Shiow Jan, Yun-Chien Cheng

**Affiliations:** 1 Department of Mechanical Engineering, College of Engineering, National Yang Ming Chiao Tung University, Hsinchu 300, Taiwan; dichun.en11@nycu.edu.tw (D.-C.W.); q102a8.en13@nycu.edu.tw (H.-H.C.); 2 Department of Medicine, National Taiwan University Cancer Center, Taipei 106, Taiwan; 3 Department of Internal Medicine, National Taiwan University Hospital, Taipei 100, Taiwan; 4 Department of Internal Medicine, National Taiwan University Hsin-Chu Hospital, Hsinchu 300, Taiwan; 5 Department of Laboratory Medicine, National Taiwan University Hospital, National Taiwan University College of Medicine, Taipei 100, Taiwan; isjan15719@ntu.edu.tw; 6 Department of Electrical Engineering, College of Electrical Engineering and Computer Science, National Taiwan University, Taipei 106, Taiwan

**Keywords:** cytology, deep learning, endobronchial ultrasound, few-shot learning, lung cancer, pulmonary metastasis

## Abstract

**Background**: Endobronchial ultrasound (EBUS) is widely used for the diagnosis of pulmonary lesions. When combined with rapid on-site cytologic evaluation (ROSE), it can improve diagnostic accuracy and facilitate early identification of malignancy origin. However, differentiating primary lung cancer from metastatic tumors (e.g., breast or colorectal origin) during ROSE remains challenging due to limited cytopathology support and morphological similarities between tumor cells. This study aimed to develop a computer-aided diagnostic (CAD) system for classifying malignancy types from limited cytological samples to facilitate clinical decision-making. **Methods**: We utilized a retrospective dataset of cytological images obtained during EBUS procedures between November 2018 and June 2024. The study focused on classifying three malignancies: pulmonary adenocarcinoma, metastatic breast cancer, and metastatic colorectal cancer. A deep learning-based model (PLFCH) was developed, integrating few-shot learning, parameter-efficient fine-tuning, and a hybrid CNN–Transformer architecture to address limited annotated data. **Results**: A total of 41 patients with 346 cytological images were included. Under a 3-way 5-shot setting, the proposed model achieved an accuracy of 49.26%, outperforming existing few-shot learning methods, with precision, recall, and F1-score of 0.477, 0.493, and 0.480, respectively. Increasing the number of reference images to 20 per class further improved accuracy to 55.48%. **Conclusions**: The proposed framework demonstrated improved performance for cytological classification under limited data conditions. These findings provide an early proof of concept for applying few-shot learning to cytological assessment during EBUS procedures. Further improvements in model performance and validation in prospective, real-time clinical settings are required before its potential role in assisting diagnostic decision-making can be established.

## 1. Introduction

Cancer is a leading cause of death worldwide, accounting for nearly 10 million deaths annually, with metastasis responsible for the majority of cancer-related mortality [1]. The lungs are a common site of metastasis for various malignancies, partly because of their extensive vascular network and their role as a major capillary bed receiving the entire cardiac output, which facilitates the hematogenous dissemination and trapping of circulating tumor cells [2]. Accurate differentiation between primary lung cancer and pulmonary metastases is clinically critical, as these conditions differ substantially in prognosis and treatment strategies. Therefore, timely and reliable diagnosis of pulmonary lesions is essential. Endobronchial ultrasound (EBUS) is a minimally invasive technique for evaluating pulmonary and mediastinal lesions, with a favorable safety profile [3,4]. Integration with rapid on-site cytologic evaluation (ROSE) allows immediate assessment of specimen adequacy and preliminary cytological interpretation during the procedure, potentially facilitating appropriate specimen acquisition and reducing the need for repeat procedures [5,6,7]. However, ROSE implementation is limited by the requirement for on-site cytologists [5]. Although pulmonologists can perform ROSE after brief training [8,9], adoption remains low and accurate differentiation between primary lung cancer and metastatic lesions is still challenging. Therefore, a more practical and accessible approach for real-time diagnostic assessment is needed.

Artificial intelligence (AI) offers considerable potential for cytologic image analysis because deep learning models can automatically extract complex morphological features and identify subtle image patterns from cytological images [10]. However, most studies have focused on distinguishing benign from malignant lesions, with limited application to the classification of different malignant cell types [11]. In addition, obtaining sufficiently large and accurately annotated datasets for individual malignant subtypes is challenging, further limiting the development of subtype-specific models [10]. Robust deep learning models typically require large annotated datasets to learn representative and generalizable features; when training data are limited, the risk of overfitting and poor generalization to unseen cases increases [12]. In clinical practice, pulmonary lesions in patients with known extrapulmonary malignancies may be considered metastatic based on clinical and radiological findings, and histopathological confirmation is therefore not always pursued [13]. Consequently, fewer cytological specimens from pulmonary metastases are available for image acquisition and pathological annotation, limiting the development of large, well-annotated datasets for AI training.

Few-shot learning (FSL) addresses data scarcity in medical image classification [14,15,16], with advances in meta-learning, multimodal learning, and feature representation [17,18,19,20,21]. Nevertheless, these methods often show limited generalizability to real-world clinical data characterized by class imbalance, domain shift, and subtle inter-class differences—particularly between primary lung cancer and metastatic tumors. To address these challenges, recent approaches incorporate parameter-efficient fine-tuning (PEFT), fine-grained image classification (FGIC), hybrid CNN–Transformer architectures, and contrastive learning to improve adaptability and generalization [22,23,24,25,26]. Despite these advances, AI-based approaches specifically designed for cytological differentiation between primary lung cancer and pulmonary metastases, particularly using cytological images obtained during EBUS procedures, remain limited. To bridge this gap, we propose a deep learning-based computer-aided diagnostic system that integrates these techniques to improve classification performance under real-world clinical constraints. Unlike previous approaches primarily evaluated on relatively large benchmark datasets [17,18,19,20,21], the proposed framework is tailored to data-scarce and imbalanced cytological datasets with substantial domain shift and subtle morphological differences between malignant tumor types. Few-shot learning enables learning from limited samples, PEFT facilitates adaptation to the target cytology domain, fine-grained feature learning captures subtle discriminative morphology, and the hybrid CNN–Transformer architecture combines local and global representations; contrastive learning further enhances feature discriminability and generalization. From a research perspective, this integrated framework provides an approach for fine-grained cytological classification under limited-data conditions and represents an early step toward AI-assisted cytological interpretation during EBUS procedures. The main contributions are as follows:

1. A computer-aided diagnostic system for rapid cytologic differentiation between primary lung cancer and pulmonary metastases.
2. A few-shot learning framework tailored for imbalanced and data-scarce clinical settings.
3. An integrated model combining parameter-efficient fine-tuning, fine-grained feature learning, hybrid CNN–Transformer architecture, and contrastive learning to improve classification performance.

## 2. Materials and Methods

### 2.1. Participants and Data Acquisition

We retrospectively collected data from patients who underwent EBUS procedures at the National Taiwan University Cancer Center and National Taiwan University Hospital, Hsin-Chu Branch, between November 2018 and June 2024. The study was approved by the Institutional Review Board of the National Taiwan University Cancer Center (IRB# 202407027RINA), with a waiver of informed consent due to de-identified data. All procedures complied with the Declaration of Helsinki.

For peripheral pulmonary lesions, a flexible bronchoscope with a 20-MHz radial EBUS probe (UM-S20-17S; Olympus, Tokyo, Japan) was used for localization, providing high-resolution cross-sectional imaging of the target lesion, followed by transbronchial biopsy (TBB). For mediastinal or hilar lesions, convex probe EBUS (BF-UC260FW; Olympus, Tokyo, Japan) was used, and transbronchial needle aspiration (TBNA) was performed. Both peripheral pulmonary and mediastinal/hilar lesions were included because diagnostic cytological specimens could be obtained from either site using the corresponding EBUS approach.

### 2.2. Cytology Image Acquisition and Dataset

Cytological specimens obtained via TBB or TBNA were smeared onto glass slides and stained using a rapid staining method (Hemacolor; Merck KGaA, Darmstadt, Germany) for ROSE. Hemacolor was used as the routine staining method at our institutions because its rapid staining process facilitates immediate cytological assessment during ROSE. All slides were examined on-site by pulmonologists with more than 6 months of cytology training and over 10 years of clinical experience using a microscope (BX43; Olympus, Tokyo, Japan). During routine ROSE, when diagnostically relevant cytological findings were identified, representative microscopic fields were captured at 100×, 200×, or 400× magnification using a digital camera system (DP22; Olympus, Tokyo, Japan). These images were acquired as selected microscopic fields during routine ROSE rather than through systematic whole-slide imaging.

### 2.3. Data Preprocessing

This study focuses on differentiating primary lung adenocarcinoma from pulmonary metastases originating from breast and colorectal cancers, which represent two of the most common metastatic tumor types. The clinical cytology dataset was not conventionally divided into fixed training, validation, and test sets. Instead, the Mini-ImageNet dataset [27] was used exclusively for meta-training and validation within the few-shot learning framework, from which 600 training tasks and 150 validation tasks were generated under a 3-way 5-shot configuration. The clinical cytology dataset, comprising 346 images from 41 patients, was reserved for target-domain adaptation and evaluation and was used to construct 600 testing tasks. Within each testing task, each of the three cytological classes comprised 5 support images and 15 query images. Patient cases were separated between the support and query sets within each task so that no patient contributed images to both sets. The support images were used for task-specific fine-tuning, whereas the actual query images were withheld from fine-tuning and used only for final evaluation.

Standard data augmentation techniques, including random resized cropping, horizontal flipping, color jittering, and affine transformations, were applied. In addition, two domain-specific strategies were introduced: (1) local patch cropping to emphasize cellular morphology, and (2) random masking to simulate occlusion and improve model robustness.

### 2.4. Overall Framework

**Figure 1:**
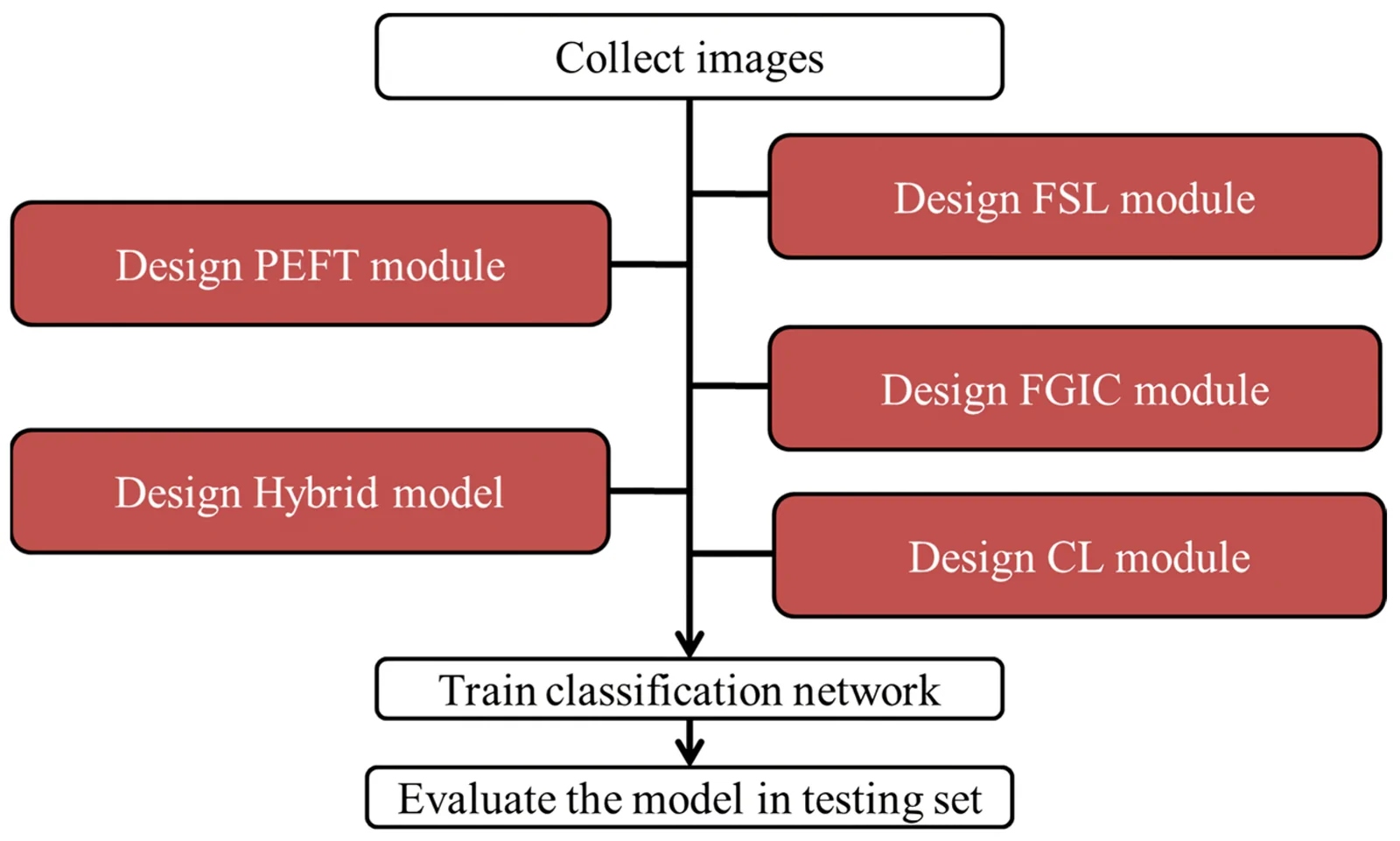
Experiment process.

The overall experimental workflow is illustrated in Figure 1. This study employs a few-shot learning framework based on Prototypical Networks [28], combined with a pre-train → meta-learn → fine-tune (PMF) strategy [17].

**Figure 2:**
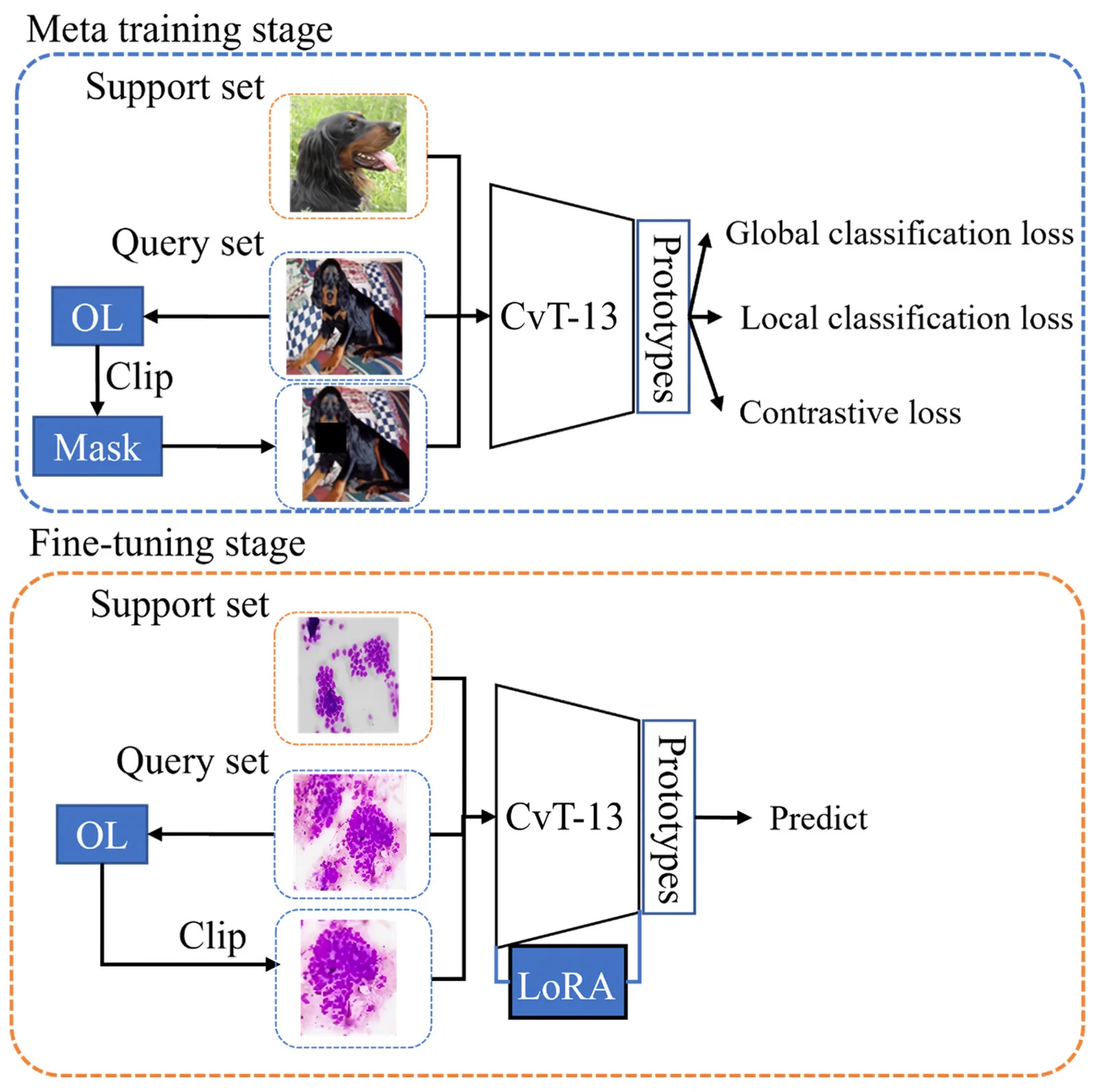
Model overview.

The proposed model, termed PLFCH, is illustrated in Figure 2; it integrates parameter-efficient fine-tuning (PEFT) using Low-rank adaptation (LoRA) [29], fine-grained feature extraction, a hybrid CNN–Transformer backbone (CvT) [30], and contrastive learning (CL) [31].

The framework consists of two stages: a meta-training stage for generalizable feature learning and a fine-tuning stage for task-specific adaptation.

### 2.5. Meta-Training Stage

**Figure 3:**
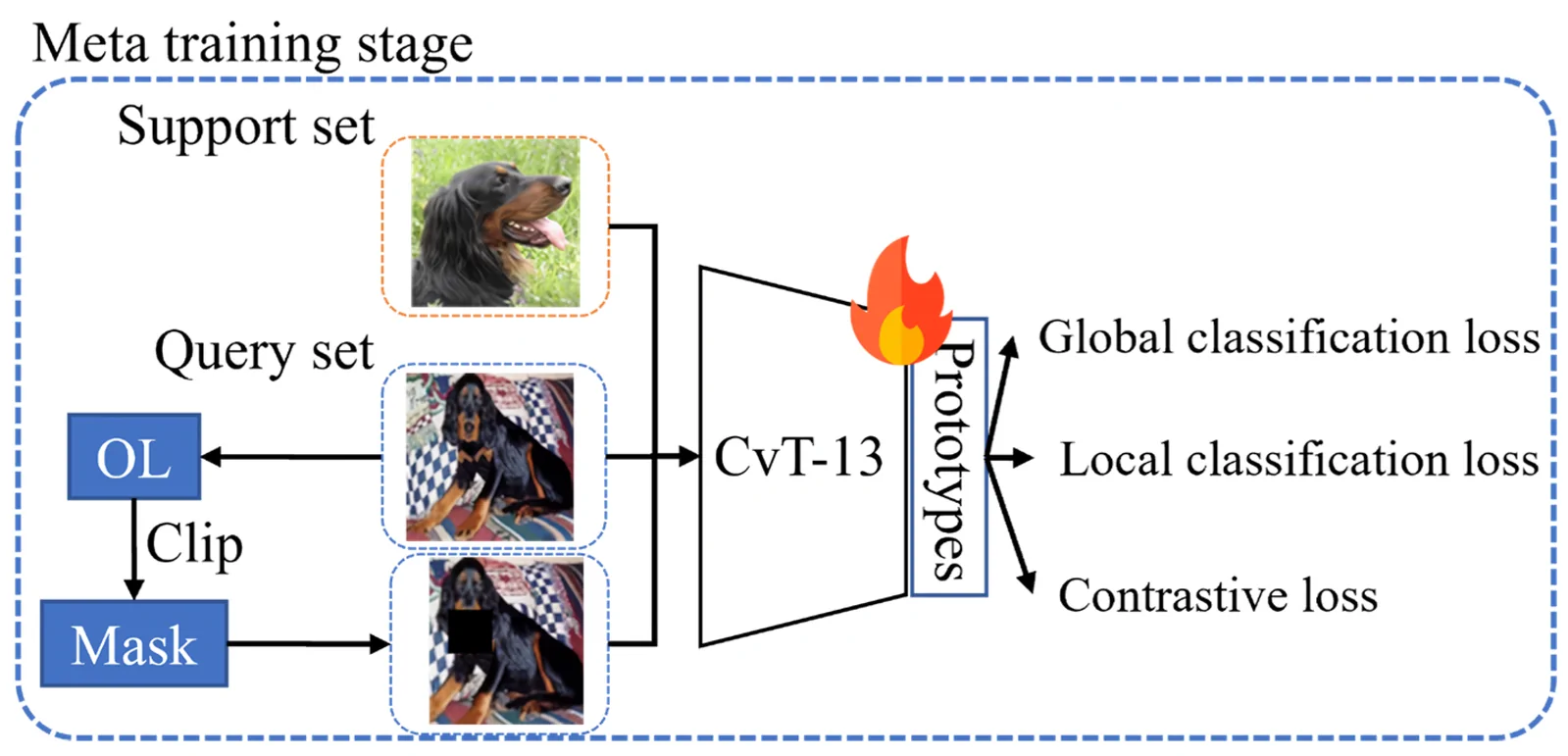
Architecture of the meta-training stage. The flame icon indicates trainable parameters.

The architecture of the meta-training stage is illustrated in Figure 3. For this phase, we utilized the Mini-ImageNet dataset rather than our cytology dataset. Since the cytology dataset contains only three categories and a limited number of clinical images, using it alone would provide insufficient task diversity for effective meta-training and substantially increase the risk of overfitting. In contrast, the greater class diversity of Mini-ImageNet enables the construction of diverse episodic tasks and facilitates the learning of more transferable feature representations. The model is trained under a 3-way 5-shot setting using Prototypical Networks [28]. During each training epoch, the model is optimized on the fixed set of 600 training tasks and subsequently evaluated on 150 validation tasks. The optimal weights yielding the highest validation accuracy across all epochs are retained for the subsequent fine-tuning stage. In this architecture, class prototypes are computed as the mean feature embeddings of support samples, and classification is performed based on the distance to these prototypes.

To enhance fine-grained feature learning, attention maps from the CvT backbone [30] are used to identify informative regions through an object location (OL) module [26]. The extracted local patches are further augmented with random masking, and a contrastive learning scheme is introduced to jointly optimize global and local representations, thereby improving feature discriminability under limited data conditions.

The training objective includes classification losses for both global and local images (cross-entropy loss) and contrastive losses to enforce representation consistency.

### 2.6. Fine-Tuning Stage

**Figure 4:**
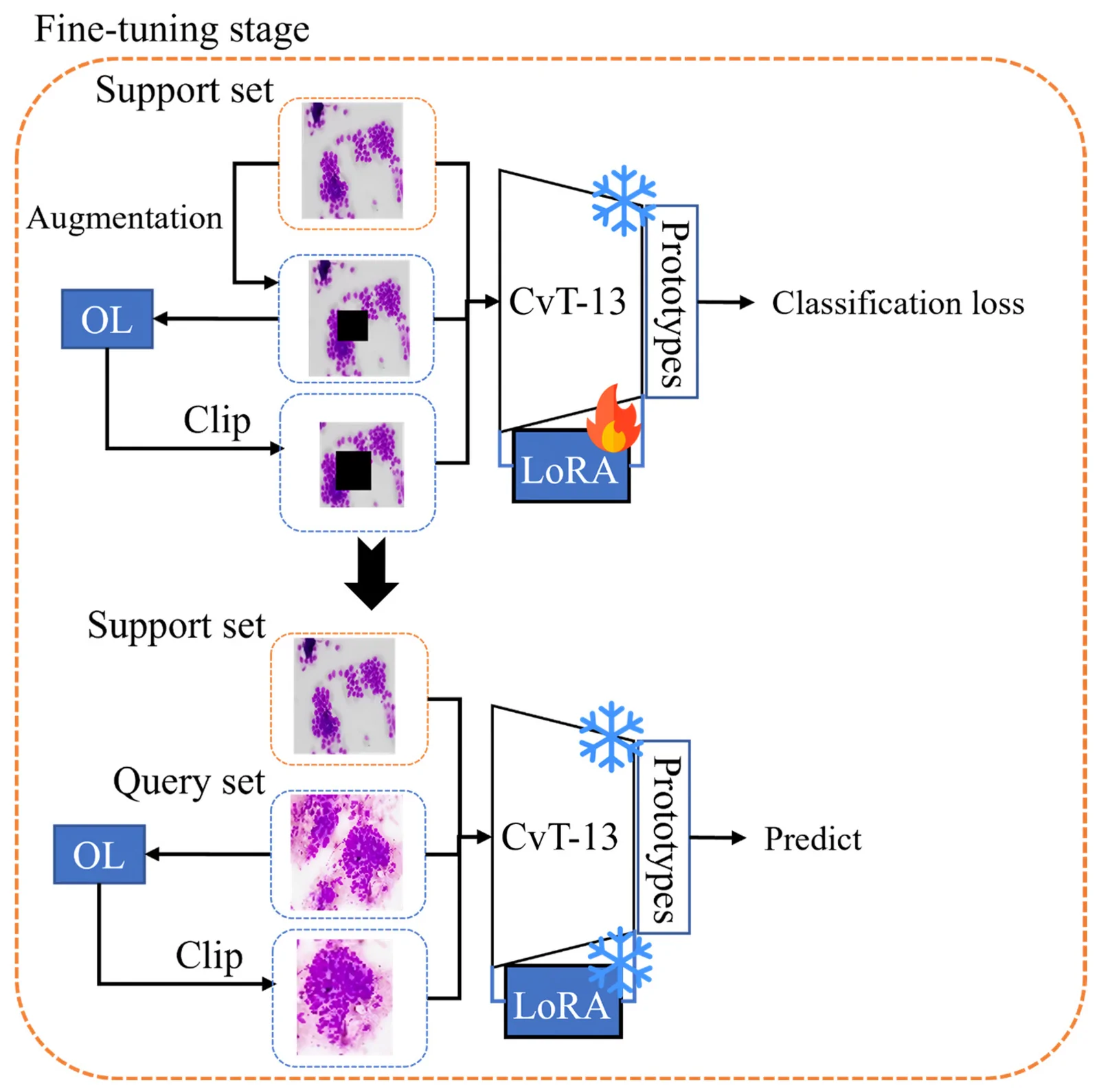
Architecture of the fine-tuning stage. The snowflake icon represents frozen parameters, whereas the flame icon indicates trainable parameters.

The fine-tuning stage is illustrated in Figure 4. In this phase, 600 testing tasks constructed exclusively from the target cytology dataset are used for target-domain adaptation and evaluation. Within each target-domain testing task, parameter-efficient fine-tuning is performed using LoRA [29] to adapt the meta-trained model to the cytological data, which updates a subset of parameters within the attention layers of the backbone network.

During fine-tuning, we first perform data augmentation on the support set to generate a pseudo-query set. This pseudo-query set is processed by the OL module to produce localized images, which are then fed into the model to extract features. The support set is used to construct a Prototypes classifier, which is subsequently used to predict both global and local versions of the pseudo-query set. The global and local prediction results are summed and compared with the labels to compute the classification loss for fine-tuning. After fine-tuning is completed, the actual query set, which is not used during fine-tuning, is input into the model for final evaluation, and the final classification result is obtained by combining global and local predictions. The overall final performance of the framework is determined by averaging the accuracy across all 600 testing tasks.

### 2.7. Implementation Details

All experiments were conducted on a workstation equipped with an Intel Core i9-13900K CPU (Intel Corporation, Santa Clara, CA, USA) and an NVIDIA GeForce RTX 4090 GPU (NVIDIA Corporation, Santa Clara, CA, USA). The model was implemented using PyTorch (version 2.0; PyTorch Foundation, Menlo Park, CA, USA), and training was performed with standard optimization settings for few-shot learning.

### 2.8. Statistical Analysis

Model performance was evaluated under a standard 3-way 5-shot configuration and averaged across 600 testing tasks. To ensure rigorous evaluation, patient cases were strictly separated between the support and query sets to prevent data leakage. The 95% confidence interval (CI) for the mean accuracy of the final PLFCH model across the 600 testing tasks was calculated as the mean ± 1.96 × standard error (SE), where SE was calculated as the standard deviation divided by the square root of the number of testing tasks. Formal statistical hypothesis testing between the proposed PLFCH and the comparative models was not performed.

The primary outcome measures included accuracy, precision, recall, and F1-score. Sensitivity analyses were conducted by varying the number of reference images (1, 5, 10, and 20 shots) to assess model robustness under different data availability conditions. All analyses were performed using Python (version 3.10; Python Software Foundation, Wilmington, DE, USA), PyTorch (version 2.0; PyTorch Foundation, Menlo Park, CA, USA), and scikit-learn (version 1.2; INRIA, Paris, France).

## 3. Results

### 3.1. Dataset Characteristics

A total of 41 patients were included in this study, with cytological images obtained from EBUS procedures using transbronchial biopsy (TBB) or transbronchial needle aspiration (TBNA). The dataset comprised images of primary lung adenocarcinoma and pulmonary metastases originating from breast and colorectal cancers.

In total, 346 cytological images were collected, including 120 images of primary lung adenocarcinoma, 84 images of colorectal cancer metastases, and 142 images of breast cancer metastases. Detailed patient characteristics and image distributions are summarized in Table 1.

### 3.2. Comparison of Backbone Networks

The performance of different backbone networks is presented in Table 2. Among all evaluated models, the CvT backbone achieved the best overall performance, with the highest accuracy (47.04%), precision (0.463), recall (0.470), and F1-score (0.466).

ResNet-18 achieved comparable performance (accuracy: 42.05%), whereas ResNet-50 and ViT-Small showed slightly lower performance. ViT-Base achieved an accuracy of 42.57%, which remained inferior to that of the CvT backbone.

### 3.3. Comparison of Fine-Tuning Methods

The performance of different fine-tuning strategies is summarized in Table 3. LoRA achieved the best overall performance, with an accuracy of 47.93%, precision of 0.474, recall of 0.479, and F1-score of 0.477, while requiring only 0.74% of trainable parameters.

BitFit achieved comparable performance (accuracy: 47.40%) with even fewer trainable parameters (0.31%). Full fine-tuning resulted in an accuracy of 47.04%, whereas fine-tuning only the final layer yielded lower performance (accuracy: 45.21%). The model without fine-tuning achieved an accuracy of 44.66%.

### 3.4. Ablation Study

The results of the ablation study are summarized in Table 4. The baseline model achieved an accuracy of 44.00%. Incorporating the CvT backbone improved accuracy to 47.04% (+3.04%). The addition of LoRA further improved performance to 47.93% (+3.93%). Incorporating the OL module resulted in an accuracy of 48.00% (+4.00%), while further integration of contrastive learning improved accuracy to 48.77% (+4.77%). The full model, integrating all components, achieved the best performance, with an accuracy of 49.26% (95% CI, 48.55–49.97%), precision of 0.477, recall of 0.493, and F1-score of 0.480.

### 3.5. Comparison with Existing Methods

A comparison with existing few-shot learning methods is presented in Table 5. The proposed PLFCH model achieved the best performance, with an accuracy of 49.26%, precision of 0.477, recall of 0.493, and F1-score of 0.480. In comparison, other methods, including ProtoNet [28], MetaOpt [35], LDPNet [21], PMF [18], and CAML [19], demonstrated lower performance across all evaluation metrics.

### 3.6. Performance Under Different Shot Settings

**Figure 5:**
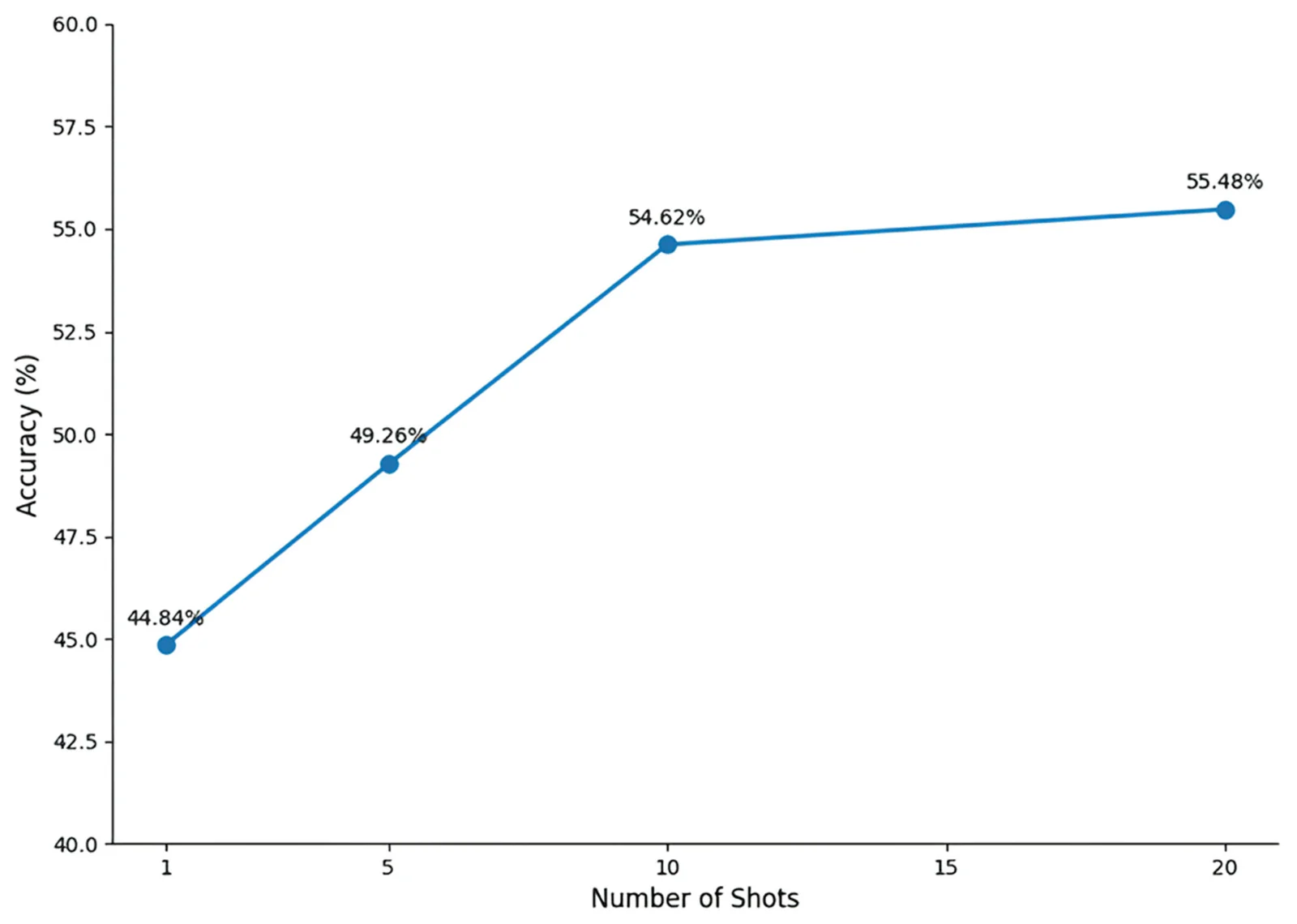
The performance under varying numbers of shots.

The performance under varying numbers of shots is illustrated in Figure 5. Accuracy increased from 44.84% (1-shot) to 49.26% (5-shot), 54.62% (10-shot), and 55.48% (20-shot). Similar trends were observed for precision, recall, and F1-score, as shown in Table 6.

## 4. Discussion

In this study, we developed a deep learning-based framework for cytological classification of pulmonary lesions, focusing on differentiating primary lung adenocarcinoma from metastatic tumors. The proposed PLFCH model outperformed existing few-shot learning methods across multiple evaluation metrics and demonstrated consistent improvements through component integration, as supported by the ablation analysis. Model performance also improved with increasing numbers of support samples, indicating effective utilization of limited data. In the prototype-based few-shot learning framework, additional support samples provide a broader representation of within-class variability and enable more representative class prototypes to be constructed, thereby improving the discrimination of query samples [28].

Previous few-shot learning approaches, including Prototypical Networks and meta-learning-based methods, have shown promising results in general image classification but often perform suboptimally in real-world medical datasets due to class imbalance, domain shift, and subtle inter-class variations [14,15,16]. Class imbalance may bias feature learning toward more frequently represented classes, whereas domain shift between meta-training data and clinical cytological images may reduce the transferability of learned representations and make adaptation to the target task more difficult [11,12,13,18]. In addition, conventional approaches rely primarily on global feature representations, which may be insufficient for distinguishing cytological images with fine-grained morphological differences [26,36,37,38]. Direct fine-tuning strategies are also prone to overfitting under limited data conditions, restricting their clinical applicability [22,23,24,25]. AI-based cytological classification has been increasingly investigated, and several studies have explored tumor-origin prediction using cytological images [39,40,41,42,43]. These studies have included cytological specimens from cerebrospinal fluid, pleural effusions, and peritoneal/ascitic fluid and were conducted using substantially different dataset scales and learning settings. In contrast, AI-based differentiation between primary lung cancer and pulmonary metastases using cytological images obtained during EBUS procedures remains largely unexplored.

**Figure 6:**
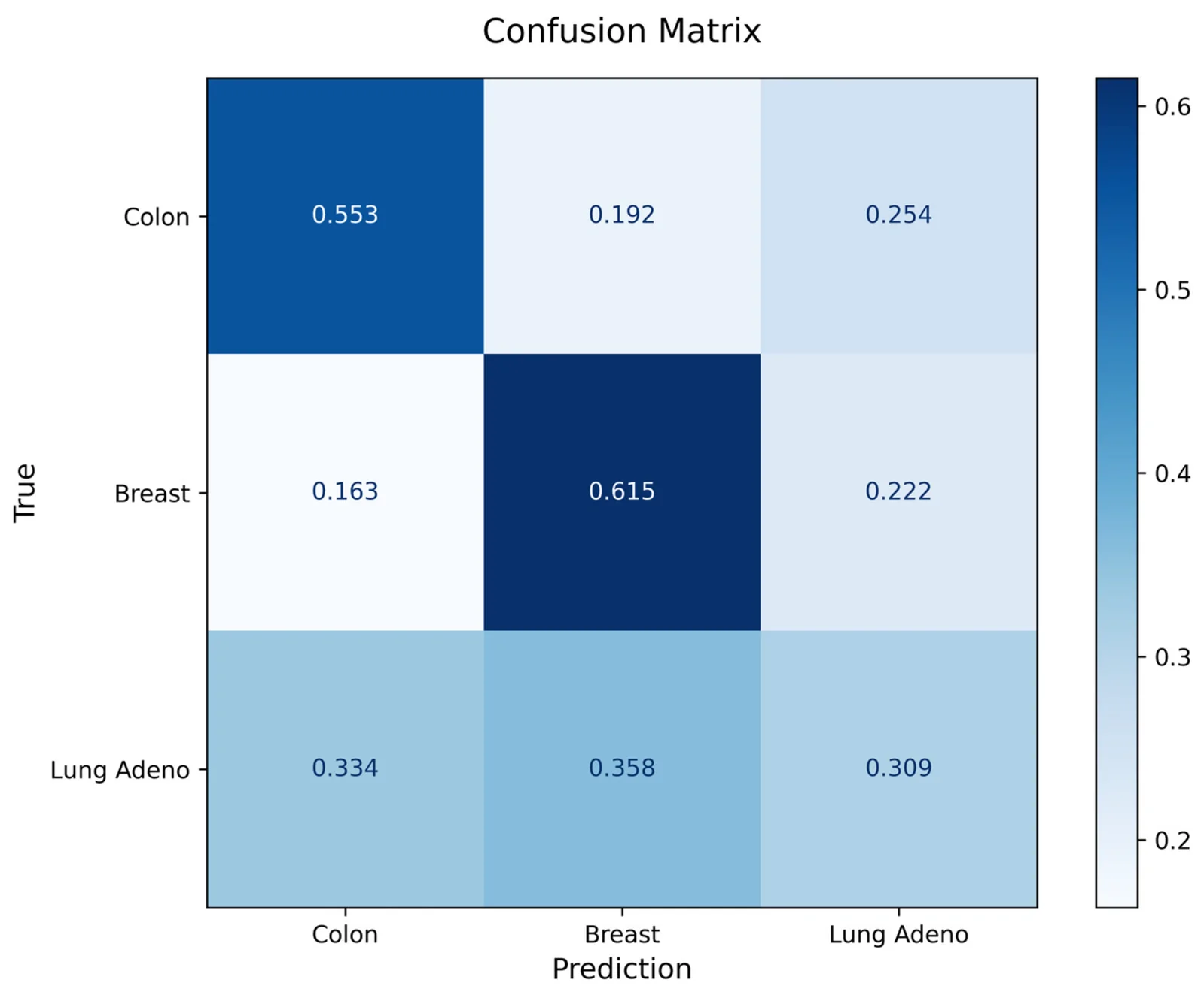
Normalized confusion matrix of the final PLFCH model evaluated across 600 testing tasks under the 3-way 5-shot setting.

In the present study, the proposed PLFCH framework achieved improved performance compared with existing methods (Table 5), reaching an accuracy of approximately 49%, whereas most comparative methods achieved accuracies below 40%. The model consistently outperformed all compared methods across multiple evaluation metrics. This improvement is likely attributable to the integration of multiple complementary components. Fine-grained feature extraction enables the model to capture subtle morphological differences between tumor types by integrating localized discriminative features with global contextual representations [26,36,37,38], while parameter-efficient fine-tuning facilitates effective adaptation under limited target data conditions [22,23,24,25]. In addition, hybrid CNN–Transformer architectures enhance feature representation by combining local and global contextual information [44,45], and contrastive learning further improves feature discriminability by separating visually similar categories [46,47]. Nevertheless, the overall accuracy of 49.26% remains modest and should not be considered sufficient for independent clinical use or real-time diagnostic support during ROSE. Rather, the present findings should be interpreted as an early proof of concept demonstrating the feasibility of few-shot learning for this challenging cytological classification task. To further understand the model’s practical limitations, we analyzed its misclassification patterns based on the confusion matrix (Figure 6). The model showed higher recall for breast metastasis and colorectal metastasis, with recall rates of 61.5% and 55.3%, respectively. In contrast, primary lung adenocarcinoma showed the lowest recall (30.9%). Among true primary lung adenocarcinoma samples, 35.8% were misclassified as breast metastasis and 33.4% as colorectal metastasis, indicating substantial confusion between primary lung adenocarcinoma and the metastatic tumor categories. These findings highlight an important limitation of the current model in distinguishing subtle morphological differences among these cytological categories.

The ablation study provides further insight into the contribution of each component (Table 4). Incorporating the CvT backbone improved accuracy from 44.00% to 47.04%, reflecting enhanced feature representation. The addition of LoRA further improved performance to 47.93%, indicating efficient adaptation with reduced overfitting risk [22,23,24,25]. Compared with full fine-tuning, LoRA updates only a small subset of parameters while preserving most pre-trained representations, which may reduce susceptibility to overfitting and facilitate adaptation under few-shot conditions. The OL module yielded additional gains (48.00%), highlighting the importance of focusing on discriminative regions, while contrastive learning further improved accuracy to 48.77%, suggesting enhanced feature separability [46,47]. Specifically, by enforcing representation consistency between the complete global images and the randomly masked local patches, this contrastive learning mechanism compels the model to learn robust and invariant features, thereby mitigating the risk of overfitting in few-shot scenarios. The stepwise improvement observed with the successive incorporation of these components suggests that each module provides an incremental and complementary contribution to model performance, with the full model achieving the best overall performance, reaching an accuracy of 49.26% (95% CI, 48.55–49.97%). The relatively narrow confidence interval suggests limited variability in accuracy across the 600 testing tasks; however, this does not eliminate the limitations associated with the small patient cohort.

The improved performance of the CvT architecture may be related to its ability to effectively combine CNN-based local feature extraction with Transformer-based global representation. This hybrid architecture may be well suited to cytological images, as it can simultaneously capture subtle morphological differences and broader cellular arrangements. We observed that ResNet-18 outperformed both ResNet-50 and ViT-Small, which may be related to its lower model complexity and reduced susceptibility to overfitting under limited-data conditions. This finding suggests that deeper or more complex models do not necessarily provide better performance when training data are limited. Although ResNet-18 was slightly inferior to ViT-Base, the global representation capability of the Transformer architecture may facilitate the identification of subtle differences among cytological images, potentially contributing to the better performance of ViT-Base.

Recent advances in few-shot learning and related techniques have further improved performance in data-limited medical image classification. Meta-learning frameworks enhance adaptation under limited data conditions [18,19,21], while parameter-efficient fine-tuning enables efficient model updating [22,23,24,25]. In the present study, the competitive performance of BitFit despite its small number of trainable parameters may reflect its ability to preserve pre-trained representations while allowing limited task-specific adaptation, thereby reducing the risk of overfitting under data-scarce conditions. Fine-grained image classification improves discrimination between visually similar categories [26,36,37,38], and hybrid CNN–Transformer architectures and contrastive learning further enhance feature representation and generalization [44,45,46,47].

In this study, we integrate these complementary strategies into a unified framework, which likely contributes to the observed performance improvements. This design enables the model to better address key challenges in cytological image analysis, including subtle inter-class differences and limited data availability.

Compared with radiology- and histology-based studies, AI applications in cytological image analysis remain limited [48,49]. Few studies have specifically addressed differentiation between primary lung cancer and metastatic tumors using cytological images [50]. To our knowledge, the application of AI to cytological images obtained during EBUS procedures, particularly in the context of ROSE, remains largely unexplored. This gap may be attributed to the inherent challenges of cytological data, including morphological variability, limited sample size, and annotation complexity [51]. Our study addresses this underexplored yet clinically relevant problem using a few-shot learning-based framework.

In clinical practice, ROSE during bronchoscopy requires rapid interpretation and can be challenging even for experienced cytologists [5,6,7]. It may also prolong procedure time or disrupt workflow. AI-assisted cytological interpretation may ultimately have the potential to reduce operator dependency and improve diagnostic consistency by providing standardized and reproducible image-based assessments; however, the performance observed in the present study is not yet sufficient to establish such clinical utility. Building on our previous work [52], the integration of ROI-guided NTS-Net enables rapid localization of diagnostically relevant regions and facilitates subtype classification from ROSE images. The present framework therefore represents an early step toward AI-assisted cytological interpretation during EBUS procedures, particularly in settings with limited cytopathology support, where immediate expert cytological interpretation may not always be available. Its potential clinical value will require further improvement in model performance and prospective real-time validation.

To investigate whether domain-relevant data could mitigate domain shift, we compared the pre-training efficacy of Mini-ImageNet with a custom medical dataset (combining datasets from [53] and [54] into 8 classes for training) under consistent experimental parameters (Table 7). Contrary to expectations, Mini-ImageNet yielded superior performance. We attribute this to its high category diversity, which enables the model to learn more robust and transferable features. Although the medical dataset is closer in domain, its limited variability likely restricted the model’s generalization ability. Furthermore, evaluation on public datasets showed strong performance on Mini-ImageNet and competitive results on other datasets (Table 8). While yielding an in-domain accuracy of 99.53%, the model ranks second or third across out-of-domain datasets (CUB [55], ChestX [56], ISIC [57], EuroSAT [58], CropDiseases [59]). This indicates that although its cytology-specific optimization slightly limits broader generalization, PLFCH remains highly competitive, maintaining an average rank of second and falling within 1% to 3% of the state-of-the-art models.

Several limitations should be acknowledged. First, the dataset was relatively small and derived from a single center, comprising 346 cytological images from only 41 patients. The limited number of patients may not adequately represent the biological and morphological heterogeneity encountered in routine clinical practice. Furthermore, the small sample size may increase variability in performance estimates and reduce the stability of the observed results. All cytological images were prepared using Hemacolor staining. Variations in staining protocols and image acquisition conditions across institutions may affect generalizability. Importantly, the model has not undergone external validation, as both model development and evaluation were performed using data from the same institution. Therefore, the performance of the model across different institutions and patient populations remains uncertain, and external validation using independent datasets from other institutions, preferably within larger multicenter studies, is warranted before clinical implementation. Second, only a limited number of cancer types were included. Third, annotations were based on expert interpretation, which may introduce inter-observer variability. Furthermore, the model has not yet been validated in real-world clinical settings. The present study was based on static image classification, and its ability to provide real-time assistance during EBUS procedures remains to be determined. In addition, the model was trained on cropped image regions rather than full-field ROSE images, limiting direct comparison with real-time clinical interpretation. Future studies should include a broader range of cancer types and focus on full-field ROSE image or video-based analysis, together with prospective real-time clinical validation, to determine the model’s performance and feasibility within routine clinical workflows. Such validation is necessary to determine whether the performance observed in the present retrospective image-based study can be reproduced during actual bronchoscopic procedures and whether AI assistance can be reliably integrated into real-time clinical decision-making.

## 5. Conclusions

The proposed PLFCH framework demonstrated improved performance in differentiating primary lung cancer from metastatic tumors compared with existing methods. This improvement is likely attributable to the integration of fine-grained feature extraction, parameter-efficient fine-tuning, and contrastive learning. However, given the modest overall accuracy, limited dataset size, and lack of real-time clinical validation, the present findings should be considered an early proof of concept rather than evidence of immediate clinical applicability. Future studies will focus on expanding dataset diversity, improving model performance, and validating the model in prospective clinical settings before its potential role in assisting diagnostic decision-making during bronchoscopy can be established.

## Figures and Tables

**Table 1:** Characteristics of the study population and cytological image dataset.

Characteristics	Total	Lung Cancer	Colon Cancer	Breast Cancer
**Patients**				
Number	41	12	9	20
Age (years old, range)	69.2 (35–92)	73.8 (54–82)	65.1 (52–84)	68.2 (35–92)
Sex (male, %)	10 (24.4)	4 (33.3)	6 (66.7)	0 (0)
TBB (***n***, %)	23 (56.1)	7 (58.3)	5 (55.6)	11 (55)
TBNA (***n***, %)	9 (22.0)	1 (8.3)	3 (33.3)	5 (25)
TBB + TBNA (***n***, %)	9 (22.0)	4 (33.3)	1 (11.1)	4 (20)
**Cytological images (** ***n*** **, %)**				
Total number	346	120 (34.7)	84 (24.3)	142 (41.0)

*n*, number; TBB, transbronchial biopsy; TBNA, transbronchial needle aspiration.

**Table 2:** Comparison of classification performance across different backbone networks.

Backbone (Parameters)	Accuracy (%)	Average Precision	Average Recall	Average F1-Score
Resnet18 (11.7 M) [32]	42.05%	0.410	0.421	0.415
Resnet50 (25.6 M) [32]	41.83%	0.403	0.418	0.410
ViT-small (22 M) [33]	38.46%	0.386	0.385	0.386
ViT-base (85 M) [34]	42.57%	0.401	0.426	0.413
CvT (19.6 M) [30]	**47.04%**	**0.463**	**0.470**	**0.466**

Bold values indicate the maximum values.

**Table 3:** Comparison of classification performance across different fine-tuning strategies.

Method	Trainable Params (%)	Accuracy (%)	Average Precision	Average Recall	Average F1-Score
No fine-tuning	-	44.66%	0.435	0.444	0.439
Fine-tuning	19.61 M (100%)	47.04%	0.463	0.470	0.466
Fine-tuning*	1.79 M (9.11%)	45.21%	0.441	0.452	0.447
BitFit	**0.06 M (0.31%)**	47.40%	0.471	0.474	0.472
LoRA	0.15 M (0.74%)	**47.93%**	**0.474**	**0.479**	**0.477**

“Fine-tuning” indicates full-model fine-tuning, whereas “Fine-tuning*” denotes fine-tuning of the final layer only. Bold values indicate the maximum values.

**Table 4:** Ablation study of the proposed method.

CvT	LoRA	OL	CL	Accuracy (%)	Average Precision	Average Recall	Average F1-Score
				44.00%	0.440	0.440	0.440
✓				47.04%	0.463	0.470	0.466
✓	✓			47.93%	0.474	0.479	0.477
✓	✓	✓		48.00%	0.462	0.480	0.471
✓		✓	✓	48.77%	0.471	0.488	0.475
✓	✓	✓	✓	**49.26%**	**0.477**	**0.493**	**0.480**

Bold values indicate the maximum values. CvT, Convolutional vision Transformer; LoRA, low-rank adaptation; OL, object localization; CL, contrastive learning.

**Table 5:** Comparison of classification performance across different few-shot learning models.

Model	Accuracy (%)	Average Precision	Average Recall	Average F1-Score
ProtoNet [28]	33.73%	0.337	0.337	0.337
MetaOpt [35]	34.94%	0.351	0.349	0.350
LDPnet [21]	36.75%	0.373	0.373	0.373
PMF [18]	41.72%	0.417	0.417	0.417
CAML [19]	32.95%	0.319	0.330	0.324
PLFCH (Ours)	**49.26%**	**0.477**	**0.493**	**0.480**

Bold values indicate the maximum values.

**Table 6:** The performance under varying numbers of shots.

Shots	Accuracy (%)	Average Precision	Average Recall	Average F1-Score
1	44.84%	0.450	0.449	0.449
5	49.26%	0.477	0.493	0.480
10	54.62%	0.536	0.546	0.541
20	55.48%	0.556	0.555	0.555

**Table 7:** Impact of training data sources on model performance.

Method	Accuracy (%)	Average Precision	Average Recall	Average F1-Score
Mini-imagenet [27]	**49.26%**	**0.477**	**0.493**	**0.480**
Medical Imaging [53,54]	47.05%	0.450	0.470	0.460

Bold values indicate the maximum values.

**Table 8:** Comparison of state-of-the-art few-shot learning methods on public datasets.

Method	In Domain	Out of Domain				
	Mini-ImageNet [27]	CUB [55]	ChestX [56]	ISIC [57]	EuroSAT [58]	CropDiseases [59]
ProtoNet [28]	50.50%	68.79%	24.05%	39.57%	73.29%	79.72%
MetaOpt [35]	80.00%	41.20%	22.53%	36.28%	64.44%	68.41%
LDPnet [21]	-	70.39%	26.67%	48.06%	82.01%	89.40%
PMF [18]	74.69%	85.04%	**27.27%**	**50.12%**	**85.98%**	**92.96%**
CAML [19]	98.6%	**97.10%**	22.2%	-	77.00%	-
PLFCH (Ours)	**99.53%**	94.62%	25.07%	47.52%	84.06%	91.43%

Bold values indicate the maximum values.

## Data Availability

The data supporting the findings of this study are available from the corresponding author upon reasonable request.

## Abbreviations

The following abbreviations are used in this manuscript:

EBUS	Endobronchial ultrasound
ROSE	Rapid on-site cytologic evaluation
CAD	Computer-aided diagnostic
FSL	Few-shot learning
PEFT	Parameter-efficient fine-tuning
FGIC	Fine-grained image classification
TBNA	Transbronchial needle aspiration
LoRA	Low-rank adaptation
CL	Contrastive learning
OL	Object location
